# Editorial for the Special Issue on Heat Transfer and Fluid Flow in Microstructures

**DOI:** 10.3390/mi17020203

**Published:** 2026-02-02

**Authors:** Xuan Zhang, Steven Wang, Jin Yao Ho, Bingqiang Ji, Long Zhang

**Affiliations:** 1 Department of Energy and Power Engineering, School of Mechanical Engineering, Beijing Institute of Technology, Beijing 100081, China; 2 Department of Mechanical Engineering, City University of Hong Kong, Hong Kong 999077, China; 3 School of Mechanical and Aerospace Engineering, Nanyang Technological University, Singapore 639798, Singapore; 4 School of Astronautics, Beihang University, Beijing 100191, China

## 1. Introduction to the Special Issue “Heat Transfer and Fluid Flow in Microstructures”

By constructing precise micro-/nano-scale topological features on solid interfaces, microstructured surface technology offers a revolutionary approach to actively regulating flow and heat transfer processes [1,2,3,4]. It overcomes the performance limitations of conventional smooth surfaces, transforming passive interfaces into active functional units and thereby enabling precise control over fluid dynamics, phase change behavior, and energy transport. This technology plays a critical role in fields such as high-efficiency cooling of electronic chips [5], aerospace thermal protection [6], biomedical microfluidics [7], and enhanced heat transfer in energy systems [8]. Its core problems lie in endowing surfaces with novel physical functions and system-level performance through structural design.

The strategic significance of this field extends beyond improving local heat and mass transfer efficiency; it also reveals and exploits a series of unique physical mechanisms dominated by microscopic surface geometry. These mechanisms include inducing controlled flow separation and vortex generation through designed micro-ribs, dimples, and other features [9,10,11] to enhance mixing and convection; precisely regulating the pinning and slipping behaviors of the solid–liquid–gas three-phase contact line [12] for active management of wettability and phase change processes; and utilizing interfacial forces such as capillary forces and Marangoni stresses generated by microstructures to drive or stabilize fluid motion. A deeper understanding and wider application of these mechanisms can allow microstructured surfaces to become a key enabling technology in addressing engineering challenges such as high heat flux dissipation, enhanced phase change heat transfer, flow drag reduction, and surface anti-icing/anti-corrosion.

However, translating the significant potential of microstructured surfaces into stable, reliable, and scalable engineering applications involves complex multi-scale and multi-physics coupling challenges [13]. This requires research not only to establish structure–performance relationships between surface topography and local heat/mass transfer characteristics at the microscale but also to evaluate overall thermal performance, mechanical durability, and long-term service stability at the macroscale system level. Specific scientific issues include the optimal design and performance prediction of microstructure shape, size, and arrangement; flow and heat transfer behavior of complex fluids (e.g., non-Newtonian fluids, nanofluids) over structured surfaces [14]; retention and failure mechanisms of structural functions in multi-phase flow environments [15]; and efficient, low-cost manufacturing processes and integration technologies for large-scale microstructure fabrication [16,17].

Therefore, research on microstructured surfaces has become a comprehensive interdisciplinary frontier integrating surface engineering, micro-/nano-fabrication, fluid mechanics, heat transfer, and materials science. The ultimate goal is to achieve “designed functions” through “designed surfaces”, driving innovation in next-generation high-performance thermal management devices, efficient energy conversion systems, advanced microfluidic chips, and aerospace power systems. This calls for full-chain innovation spanning fundamental mechanisms, material systems, manufacturing processes, and system integration, achieving deep synergy among structure and function, material and process, and design and application.

**Figure 1:**
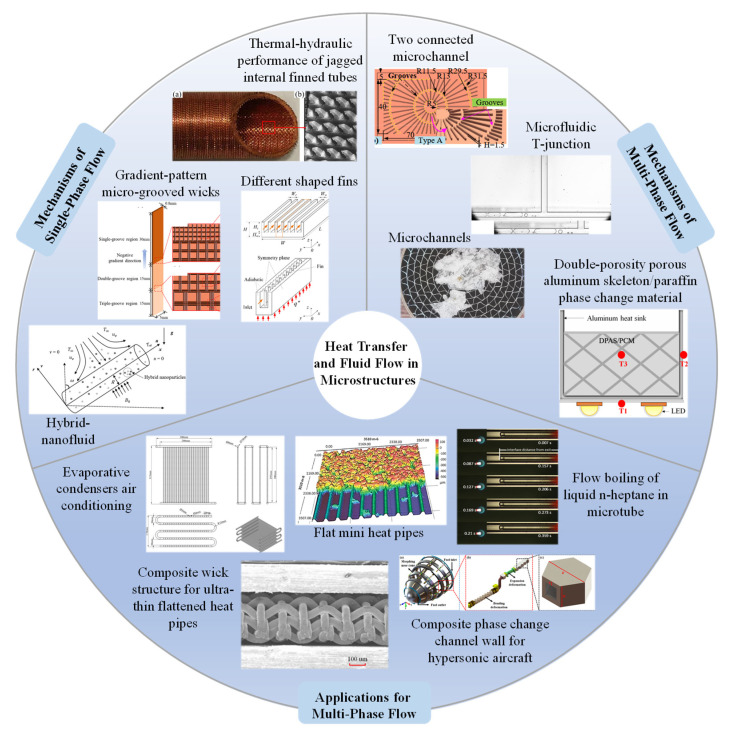
Topics covered in the Special Issue titled “Heat Transfer and Fluid Flow in Microstructures”.

This Special Issue focuses on this interdisciplinary field, bringing together a range of research achievements covering fundamental studies, numerical simulations, experimental characterizations, and application explorations. Based on their research directions and content characteristics, the contributions can be grouped into the following three topics: fundamental studies on the mechanisms of single-phase flow [contributions 4, 5, 13–15], fundamental studies on the mechanisms of multi-phase flow [contributions 1, 6, 8, 10, 11], and extended research on the applications for multi-phase flow [contributions 2, 3, 7, 9, 12], as illustrated in Figure 1. This classification framework systematically presents a complete research spectrum, from fundamental flow principles to engineering solutions.

Overall, this Special Issue reflects the latest advances in microstructured surface technology in both fundamental research and engineering applications. It not only enhances our understanding of the flow and heat transfer mechanisms influenced by micro-/nano-structures but also provides theoretical guidance and technical solutions for the design and system integration of functional surfaces orientated toward practical needs. Future research is expected to further advance toward intelligent responsive surfaces, multifunctional integrated structures, cross-scale synergistic design, and reliable applications in extreme environments, continuously driving this technology toward a more efficient, reliable, and intelligent future.

## 2. Mechanisms of Single-Phase Flow

This section explores the enhancement of single-phase heat transfer through engineered microstructures and advanced fluids. Key studies investigate the performance of novel 3D jagged finned tubes [contribution 4], laser-fabricated capillary-gradient wicks [contribution 5], liquid metal-cooled wavy microchannels [contribution 13], hybrid nanofluids in shaped tube exchangers [contribution 14], and magneto-convective hybrid nanofluid flow [contribution 15]. Collectively, they demonstrate how deliberate structural design and fluid engineering can disrupt flow, augment mixing, and significantly improve thermal transport.

## 3. Mechanisms of Multi-Phase Flow

This section delves into the role of microstructure in governing phase change processes. Research examines thermal management using gradient porosity phase change materials [contribution 1], flow boiling pattern evolution in T-shaped microchannels [contribution 6], the pseudo-desublimation of AdBlue in SCR systems [contribution 8, 11], and the performance of interconnected microchannel heat sinks [contribution 10]. These works highlight how micro-scale geometry and system architecture critically influence boiling dynamics, flow stability, and overall thermal performance in multi-phase systems.

## 4. Applications for Multi-Phase Flow

This section bridges fundamental research and practical thermal management solutions. It covers the development of composite wicks for ultra-thin heat pipes [contribution 2], bio-inspired phase change cooling channels for hypersonic aircraft [contribution 3], evaporative condensers for CO_2_ air conditioning [contribution 7], flow boiling of fuel in microtubes [contribution 9], and vaporization in flat mini heat pipes with porous structures [contribution 12]. These studies provide actionable design insights and validate performance in high-heat-flux cooling applications in electronics, aerospace, and energy systems.

To conclude, we would like to acknowledge all the authors for their contributions to the success of this Special Issue, “Heat Transfer and Fluid Flow in Microstructures”, as well as the reviewers, whose feedback helped to improve the quality of the published papers.
